# Altered cellular infiltration and cytokine levels during early *Mycobacterium tuberculosis sigC *mutant infection are associated with late-stage disease attenuation and milder immunopathology in mice

**DOI:** 10.1186/1471-2180-8-151

**Published:** 2008-09-17

**Authors:** Khairul-Bariah Abdul-Majid, Lan H Ly, Paul J Converse, Deborah E Geiman, David N McMurray, William R Bishai

**Affiliations:** 1 Center for Tuberculosis Research, Division of Infectious Diseases, Department of Medicine, Johns Hopkins University, Baltimore, MD 21231-1001, USA; 2 Department of Microbial and Molecular Pathogenesis, Texas A&M University System Health Science Center, College Station, Texas, 77843-1114, USA; 3 Aeras Global TB Vaccine Foundation, 1405 Research Boulevard, Rockville, MD 20850, USA

## Abstract

**Background:**

Mouse virulence assessments of certain *Mycobacterium tuberculosis *mutants have revealed an immunopathology defect in which high tissue CFU counts are observed but the tissue pathology and lethality are reduced. *M. tuberculosis *mutants which grow and persist in the mouse lungs, but have attenuated disease progression, have the immunopathology (*imp*) phenotype. The antigenic properties of these strains may alter the progression of disease due to a reduction in host immune cell recruitment to the lungs resulting in disease attenuation and prolonged host survival.

**Results:**

In this study we focused on the mouse immune response to one such mutant; the *M. tuberculosis *Δ*sigC *mutant. Aerosol infection of DBA/2 and SCID mice with the *M. tuberculosis *Δ*sigC *mutant, complemented mutant and wild type strain showed proliferation of mutant bacilli in mouse lungs, but with decreased inflammation and mortality in DBA/2 mice. SCID mice shared the same phenotype as the DBA/2 mice in response to the Δ*sigC *mutant, however, they succumbed to the infection faster. Bronchoalveolar lavage (BAL) fluid analysis revealed elevated numbers of infiltrating neutrophils in the lungs of mice infected with wild type and complemented Δ*sigC *mutant strains but not in mice infected with the Δ*sigC *mutant. In addition, DBA/2 mice infected with the Δ*sigC *mutant had reduced levels of TNF-α, IL-1β, IL-6 and IFN-γ in the lungs. Similarly, there was a reduction in proinflammatory cytokines in the lungs of SCID mice. In contrast to the mouse model, the Δ*sigC *mutant had reduced initial growth in guinea pig lungs. A possible mechanism of attenuation in the Δ*sigC *mutant may be a reduction in neutrophilic-influx in the alveolar spaces of the lungs, and decreased proinflammatory cytokine secretion. In contrast to mouse data, the *M. tuberculosis *Δ*sigC *mutant proliferates slowly in guinea pig lungs, a setting characterized by caseating necrosis.

**Conclusion:**

Our observations suggest that the immunopathology phenotype is associated with the inability to trigger a strong early immune response, resulting in disease attenuation. While macrophages and T cells have been shown to be important in containing *M. tuberculosis *disease our study has shown that neutrophils may also play an important role in the containment of this organism.

## Background

According to the latest WHO fact sheet, tuberculosis (TB) causes about 2 million deaths every year and 2 billion of the world's population is infected with *Mycobacterium tuberculosis *[1,2]. TB co-infection is also the number one killer of HIV patients which implicates the importance of a healthy immune system in controlling TB. *M. tuberculosis *is transmitted mainly by the respiratory route, and the primary site of infection is the lung. Following inhalation, these bacilli are phagocytosed by resident alveolar macrophages which recruit neutrophils, T cells and monocytes, and promote the local production of cytokines [3-5]. *In vitro, M. tuberculosis *triggers a Th1 type immune response which results in the release of TNF-α, IL-12 and IFN-γ [6,7]. These observations have been further substantiated *in vivo *using the mouse model [8-12]. While macrophages and T cells play key roles in the immune containment of *M. tuberculosis*, the role of other immune cells has received less attention. A recent study found that enhanced recruitment of neutrophils in lungs of some mouse strains is associated with increased susceptibility to *M. tuberculosis *infection [13], indicating that neutrophils may play a more important role during *M. tuberculosis *infection than previously thought.

Characterizing the exact roles for each of these components has been challenging and cytokine studies have produced conflicting results. IL-12 may be crucial in clearing *M. tuberculosis *infection in BALB/c mice but in a more resistant mouse strain, like C57BL/6, its effects are marginal [10]. IFN-γ is an important cytokine in controlling intracellular *M. tuberculosis *[8,11], but *M. tuberculosis*-infected-macrophages can secrete IL-6 which in turn prevents uninfected macrophages from responding to IFN-γ [14,15]. This negative feedback loop may enable the bacteria to persist in the host. Despite the inconsistencies, the host's immune system is a factor in determining the progression of the infection.

Antigenic properties of the different *M. tuberculosis *mutants also alter disease progression. Strains which can grow and persist in mouse lungs without eliciting severe damage have the immunopathology (*imp*) phenotype [16,17]. This phenotype results in reduced host immune cell recruitment to the lungs and prolonged host survival. Several *imp *mutants have been generated and tested in mice in order to characterize changes in the host tissues as well as in their immune responses. Some of these *imp *mutants include *sigH, sigE, sigF, sigD, whiB3 *and *dnaE2 *[16,18-23]. In the case of the *sigH *mutant, four weeks after the mice were infected, there were fewer CD4 and CD8 T cells recruited to lung tissues in comparison to mice infected with the wild type strain [16]. The number of IFN-γ and TNF-α expressing CD4 T cells in these mice was also reduced. In order to determine if the *imp *phenotype can only be expressed in immunocompetent mice, SCID mice were infected with *sigH, sigE*, and *sigD *separately [16,20,21,23]. Results from these studies indicate that the *imp *phenotype appears to be dependent on functional cell-mediated immunity which the SCID mice lack.

To determine when these mutants begin expressing their phenotype in the host tissue, gene expression studies as well as RT-PCR were carried out. *whiB3 *[19,24] and *sigH *[16] for example were found to be maximally induced during the early phase of mouse lung infection while *sigF *[22] was highly expressed in stationary growth phase and was associated with late-stage disease in mice. Most of these studies focused on bacterial survival in the host rather than the host's immune response. However, these mutants could reveal how the host immune system contains *M. tuberculosis *infection.

The RNA polymerase sigma factors of *M. tuberculosis*, such as SigC, confer DNA binding at specific promoter sites and play a role in transcription initiation. In a previous study we found that the Δ*sigC *mutant had an *imp *phenotype where the mutant persists in mouse lungs but fails to cause mortality as rapidly as the wild-type. SigC was found to be essential for lethality of *M. tuberculosis *in mice [20]. To address the question of host factors, we infected mice with the Δ*sigC *mutant and assessed cell infiltration patterns and cytokine levels during different time points after aerosol infection. We found that mice infected with the wild type *M. tuberculosis *strain had higher levels of inflammatory cytokines and infiltrating neutrophils when compared to the Δ*sigC *mutant infected mice. The same observation was also noted when SCID mice were exposed to the Δ*sigC *mutant strain. We also characterized the pattern of *M. tuberculosis *Δ*sigC *mutant infection in guinea pigs and found dramatically reduced bacterial proliferation in contrast to the mouse pattern. Our observations suggest that the *imp *phenotype is associated with the inability to trigger a strong early immune response, which may result in disease attenuation.

## Results

### *M. tuberculosis *Δ*sigC *mutant infection in immunocompetent mice

**Figure 1:**
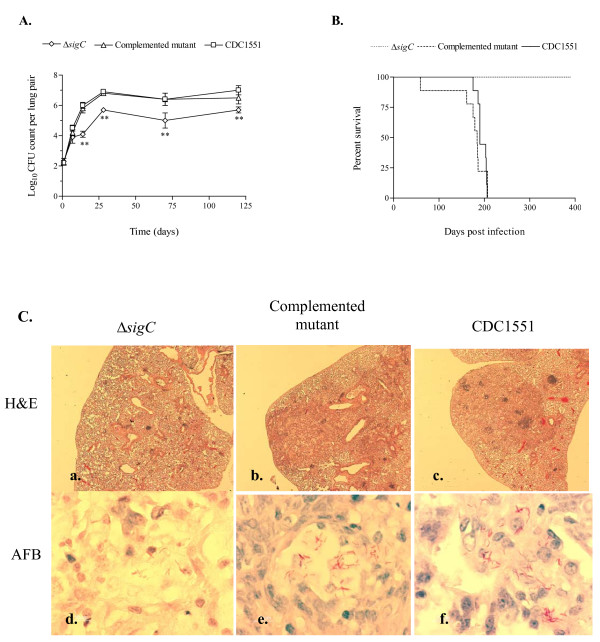
**DBA/2 mouse survival score and CFU counts**. The mean CFU counts were obtained from the lung homogenates of 5 mice per group and plated on 7H10 agar plates (A). Δ*sigC *mutant (◇), Δ*sigC *complemented mutant (△) and the wild type strain, CDC1551 (□), CFU counts were obtained by homogenizing individual lungs from each group and plating on 7H10 agar plates. Day 1 CFU counts were performed to confirm the dose of bacilli in each group. Mice were sacrificed for CFU counts and immunological assays on days 7, 14, 28, 70 and 120 post infections. CFU counts from Δ*sigC *mutant infected mice were significantly lower than in mice infected with either the CDC1551 or the complemented mutant strain (** = p < 0.01). (B) From the Kaplan-Meier survival curve, the median survival for the Δ*sigC *mutant infected group is more than 365 days post infection. This difference is significant with a log rank test p value of < 0.002. (C) Lungs of the three groups of DBA/2 mice infected with the Δ*sigC *mutant strain, the complemented Δ*sigC *mutant and the wild type strain, CDC1551, collected at day 120. Lung sections were stained with haematoxylin and eosin (H&E) (a-c) (Magnification ×20) and for acid fast bacilli (AFB) (d-f) (Magnification ×1000).

We infected three groups of mice separately with the Δ*sigC *mutant, the Δ*sigC *complemented mutant, and the wild type parental strain, CDC1551. Bacterial growth in the lungs of DBA/2 mice was observed to increase one week post infection and to plateau at week 4 for both the CDC1551 and Δ*sigC *complemented mutant groups (figure 1A). While growth of the Δ*sigC *mutant paralleled those of the wild type and complemented strains for the first week, its rate of proliferation slowed from week 2 to week 4 (figure 1A). A similar outcome was detected in an earlier study with DBA/2 mice [20]. At 28 days the Δ*sigC *mutant attained CFU counts which were 1 log unit lower than the CDC1551 and Δ*sigC *complemented mutant groups. This observation persisted up to day 120. At the same time mice exposed to the Δ*sigC *mutant remained healthy and alive for an extended time in comparison to the CDC1551 and Δ*sigC *complemented mutant groups all of which died by 200 days (figure 1B). Haematoxylin-eosin staining of lungs at day 120 from the complemented Δ*sigC *mutant and CDC1551 infected groups confirmed that these lungs had extensive internal damage in contrast to the Δ*sigC *mutant group (figure 1C; a, b and 1C). Ziehl-Neelsen staining confirmed the presence of fewer bacilli in the Δ*sigC *mutant group (figure 1C; d, e and 1f). These observations indicated that milder lung pathology, secondary to reduced bacterial growth, can contribute to host survival.

### Differential cellular infiltration in alveolar spaces seen with the Δ*sigC *mutant

**Figure 2:**
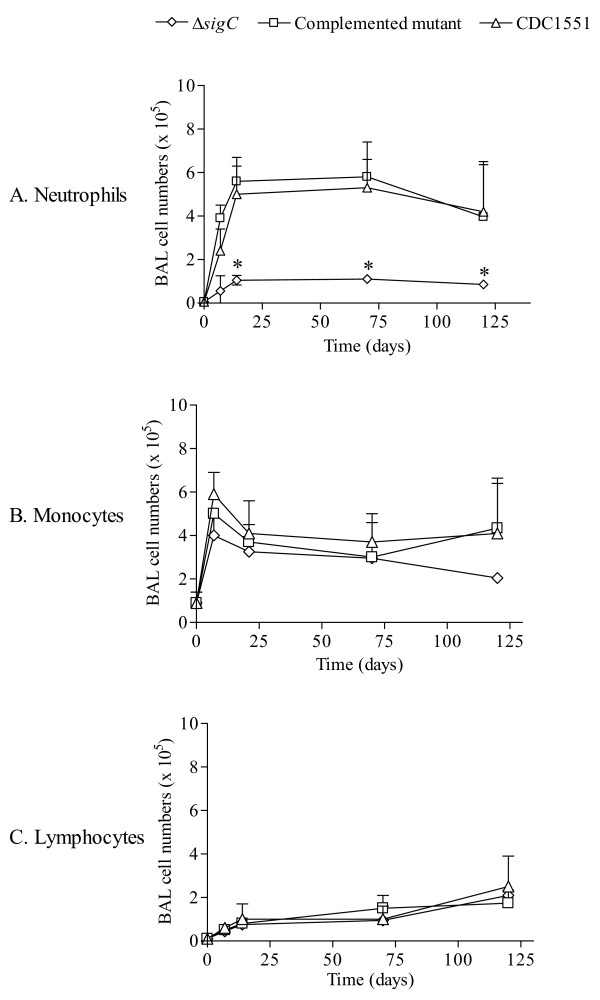
**Cell types present in the bronchoalveolar lavage (BAL) fluid of DBA/2 mice following infection with the three different *M. tb *strains**. At the time points indicated BAL fluid of each mouse was collected, counted and a sample taken for cell type determination by cytospin analysis. 100 cells were counted and differential sorting of cell types was performed blindly in triplicate. Results are expressed as the mean number of total cells harvested per mouse ± standard deviation from 5 mice per group per time points. (* = p < 0.05).

The role of resident alveolar macrophages in eliminating invading bacteria in the lung has been widely accepted [25,26]. However, less is known regarding the role of polymorphonuclear cells in pulmonary tuberculosis. Thus, we collected BAL fluid and employed cytology to classify which cell types were recruited into the alveolar spaces during disease progression in wild type versus Δ*sigC *mutant infection. The total number of recovered cells from BAL varied between less than 10^5 ^cells in non-infected mice to 1 million cells at days 14 and 70 (figure 2). Throughout this study, the number of BAL cells from mice infected with the Δ*sigC *mutant remained on average less than 5 × 10^5 ^cells per mouse in contrast to mice infected with the CDC1551 and the complemented Δ*sigC *mutant (figure 2) whose counts were ~1 × 10^6^. The lymphocyte population, which consisted of B, T and NK cells, remained at a relatively constant level throughout the study. Three cell types were detected via the cytospin assay: lymphocytes, monocytes/macrophages and neutrophils. The majority of BAL cells were alveolar macrophages at about 90%, while lymphocytes and neutrophils made up the remaining cell population. As time progressed, more neutrophils were found in the BAL fluid of both the complemented Δ*sigC *mutant and CDC1551 infected groups (figure 2). At days 14 and 70, neutrophils became the main cells in the lungs of these two groups, but this was not observed in the lungs of Δ*sigC *infected mice. Instead, decreased numbers of infiltrating neutrophils were observed at day 70 and day 120.

### Proinflammatory cytokines were reduced in lung homogenates of Δ*sigC *infected DBA/2 mice

**Figure 3:**
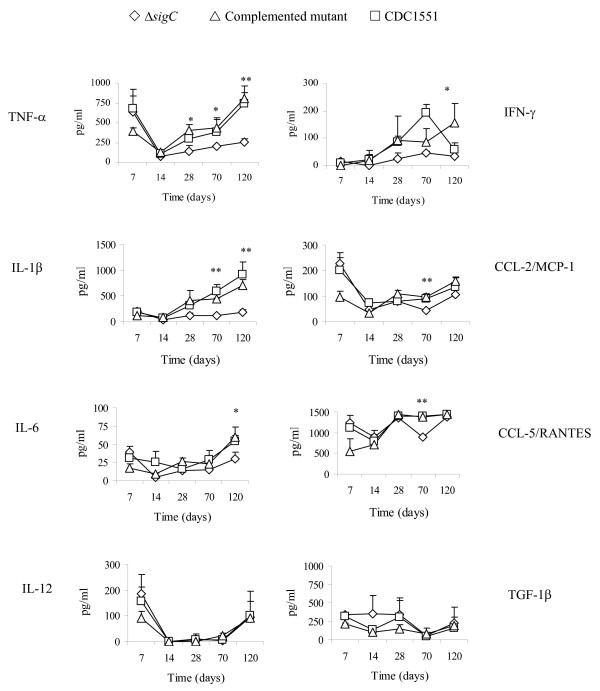
**Cytokine profile detected in the lung tissue homogenates of DBA/2 mice infected separately with the three different *M. tb *strains**. ELISA based cytokine assays were applied to determine the presence of cytokines. Supernatants were collected from lung homogenates of DBA/2 mice which were infected with Δ*sigC *mutant strain (◇), the complemented Δ*sigC *mutant (△) and the wild type strain, CDC1551 (□). Each time point represents data from 5 mice and each was performed in triplicate. (* = p < 0.05; ** = p < 0.01).

IL-1β acts on monocytes and macrophages to induce production of TNF-α, IL-6 and IL-8 to enhance bactericidal killing [27]. IL-1 β also acts on NK cells, in collaboration with IL-2 and IFN-γ. IL-1β is required for T cell dependent antibody production [28,29]. *M. tuberculosis *is a strong inducer of chemokine expression. Chemokines are involved in trafficking and recruiting cells in BAL as a means to clear the infection [30]. In mice infected with the Δ*sigC *mutant, the levels of TNF-α, IL-1β, IL-6 and IFN-γ began to drop 14 days post infection and persisted throughout the study while a significant reduction in CCL-2 and CCL-5 levels was detected 70 days after infection (figure 3). In contrast the CDC1551 and Δ*sigC *complemented mutant groups had elevated levels of TNF-α, IL-1β, IL-6, IFN-γ, as well as chemokines (CCL-2 and CCL-5) (figure 3). The IFN-γ level of the Δ*sigC *infected mice remained low throughout the infection. A sharp drop of the IFN-γ level was observed in the lung homogenates of the CDC1551 infected group at day 120. However, this difference was not statistically significant. IL-12 has been reported to play an important role in disease progression in *M. tuberculosis *infection [7,10,31], but no differences in the level of this cytokine in all three groups were observed (figure 3). TGF-1β, IL-4 and IL-10 (the latter two not shown) also exhibited the same profile indicating that these cytokines may be undetectable or inactive in the lungs during this study period.

### *M. tuberculosis *Δ*sigC *mutant infection in SCID mice

**Figure 4:**
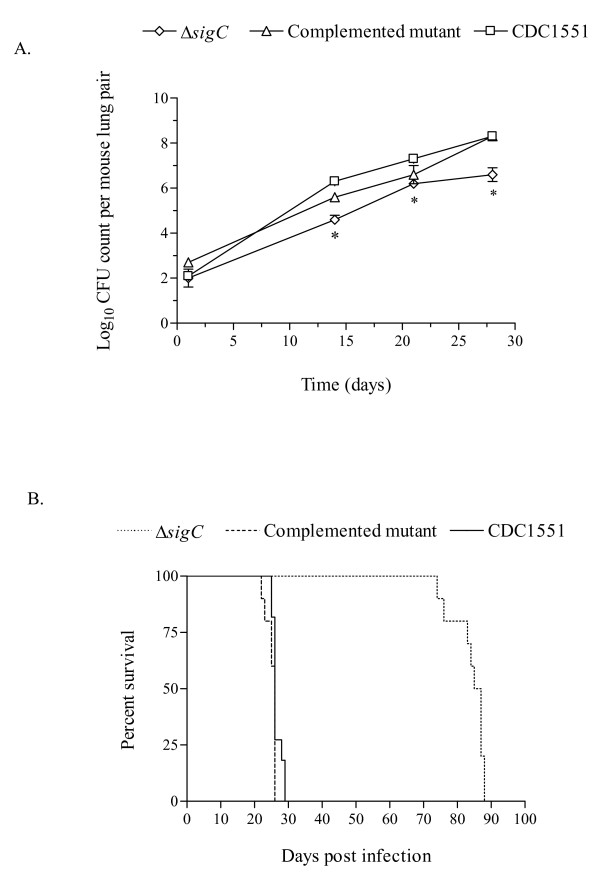
**SCID mouse survival score and lung CFU counts**. The mean CFU counts were obtained from the lung homogenates of at least 5 mice per group and plated on 7H10 agar plates (A). SCID mice received ~100 CFU per mouse via the aerosol route and these mice were sacrificed for either CFU counts or immunological assays 1, 14, 21 and 28 after infection. Day one CFU counts were performed to confirm the dose of bacilli in each group. Statistically significant differences between mice infected with the Δ*sigC *mutant strain and the CDC1551 strain were seen at days 14, 21 and 28 (* = p < 0.05). (B) From the Kaplan-Meier survival curve, the median survival for the Δ*sigC *mutant infected group is 86 days but 26 days for both the Δ*sigC *complemented mutant and the wild type strain, CDC1551. This difference is significant with a log rank test p value of < 0.002.

**Figure 5:**
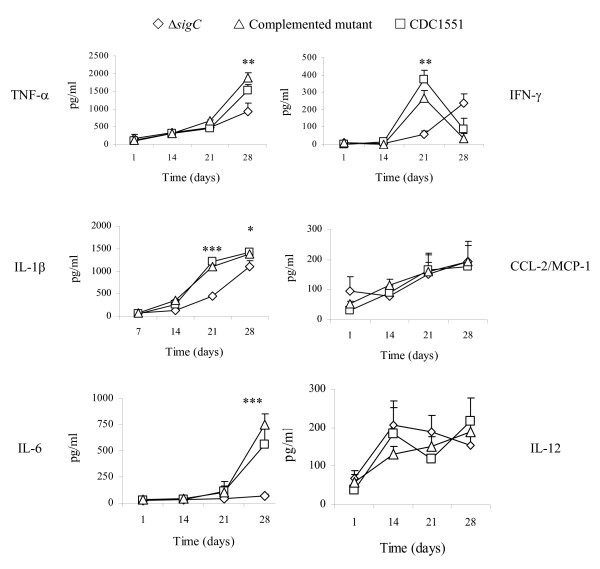
**Cytokine profiles detected in the lung tissue homogenates of SCID mice infected separately with the three different *M. tb *strains**. Supernatants were collected from lung homogenates of SCID mice which were infected with the Δ*sigC *mutant strain (◇), the Δ*sigC *complemented mutant (△) and the wild type strain, CDC1551 (□). Mice were sacrificed at days 1, 14, 21 and 28 after infection. Each time point represents data from 5 mice and each were performed in triplicate. (* = p < 0.05; ** = p < 0.01; *** = p < 0.001).

In DBA/2 mice infected with the Δ*sigC *mutant, lower numbers of infiltrating neutrophils coincided with decreased lung lesions and lower bacillary load (figure 1). Such an observation was not detected with SCID mice infected with the same mutant (data not shown). All three groups had similar profiles of the three major cell types identified (lymphocytes, monocytes/macrophages and neutrophils), where neutrophils were the dominant cell type in the BAL. The absence of a fully functional immune system would render the SCID mice vulnerable to infection in general. Thus, it was not surprising to see a gradual increase in CFU counts at day 1, 14, 21 and 28 post aerosol infection (figure 4A). At day 14, 21 and 28, a significant drop of 1 log_10 _unit in CFU counts was observed between the Δ*sigC *mutant infected mice and the CDC1551 infected group (figure 4A). Mice infected with the Δ*sigC *mutant survived 60 days longer than both the CDC1551 and Δ*sigC *complemented mutant strain infected mice (figure 4B). SCID mice infected with the Δ*sigC *mutant strain produced detectable levels of several cytokines one week after infection (figure 5). However, the level of IL-1β gradually increased and became comparable to the levels of the CDC1551 and the Δ*sigC *complemented mutant groups by day 28. A similar trend was also detected with TNF-α, IL-6 and IFN-γ, 21 days after infection. There were no differences in the levels of CCL-2 and CCL-5 (data not shown) produced by the three groups of mice. Like the DBA/2 mice (figure 3), the amount of IL-12 secreted by SCID mice was not different after infection with the three *M. tuberculosis *strains.

### Accelerated bacterial growth coincides with increased tissue damage in SCID mice

**Figure 6:**
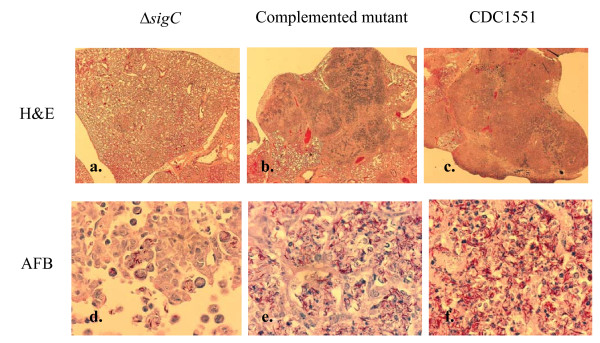
**Lung sections from SCID mice 28 days post infection**. Lung sections were stained with haematoxylin and eosin (H&E) (a-c) (Magnification ×20). Ziehl-Neelsen staining detected for the presence of acid fast bacilli (AFB) in the lungs of the three groups of infected SCID mice (d-f) (Magnification ×1000).

Lungs from the CDC1551 and the complemented mutant groups developed severe tissue damage but the damage was much milder for the Δ*sigC *mutant group. Lung sections analyzed from Δ*sigC *mutant infected mice had fewer lesions 28 days (figure 6) post infection in contrast to both the complemented mutant and CDC1551 infected groups. Ziehl-Neelsen stainings confirmed the different bacillary burden in the lungs of the three groups (figure 6). However at time of death (75–90 days post infection) the lungs of the Δ*sigC *mutant group were as severe as both the CDC1551 and complement control groups (data not shown).

### *M. tuberculosis *Δ*sigC *mutant displays a delayed growth *in vivo *(*giv*) phenotype in guinea pigs

**Figure 7:**
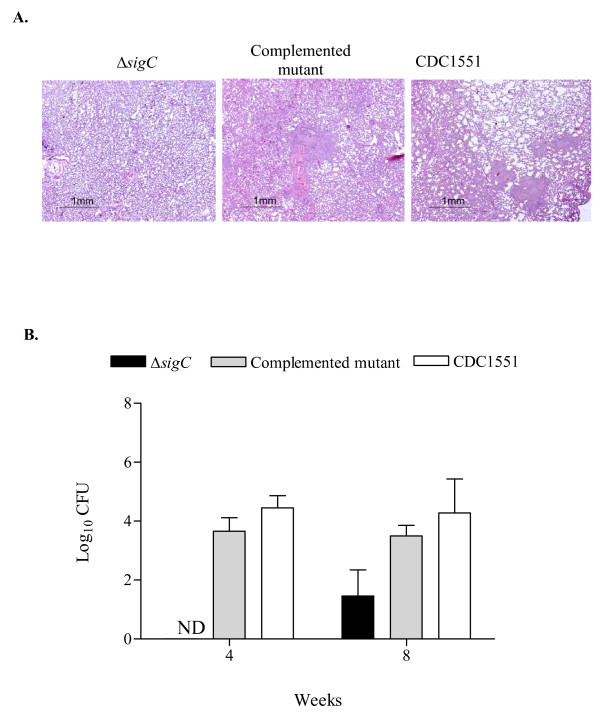
**Guinea pig lungs and CFU counts**. Lung sections of guinea pigs, 8 weeks after aerosol exposure to Δ*sigC *mutant, the complemented Δ*sigC *mutant and CDC 1551 were stained with haematoxylin and eosin (H&E) and analyzed under low power magnification (A). (Magnification ×20). Guinea pig lung CFU counts 4 weeks and 8 weeks post aerosol infection (B). On day 0, each guinea pig received ~50 to 100 CFU but day 1 CFU counts were not performed. The mean CFU counts were obtained from lung homogenates of 3 guinea pigs per group and plated on 7H10 agar plates. (ND = not detected).

*M. tuberculosis *infected guinea pigs produce well-formed caseating granulomas in their lungs similar to humans, while caseous necrosis is not routinely observed in mouse lungs infected with *M. tuberculosis *[32]. To assess the role of caseation necrosis in the survival of the *M. tuberculosis *Δ*sigC *mutant, we studied its infection pattern following aerosol inoculum in guinea pigs. Haematoxylin-eosin (HE) staining confirmed that guinea pigs infected with the Δ*sigC *mutant strain had fewer granulomas than the CDC1551 wild type and Δ*sigC *complemented mutant strains (figure 7A). Lung homogenates from guinea pigs infected with either the CDC1551 wild type strain or the Δ*sigC *complemented mutant strain showed the presence of bacterial growth in the 7H10 agar plates, 4 and 8 weeks post infection (figure 7B). However, the Δ*sigC *mutant strain was undetectable (< 100 CFU) in guinea pigs at 4 weeks and reached detectable numbers of bacteria in their lungs only at 8 weeks after infection. A similar observation was seen in their spleens (data not shown). In addition, while we were unable to keep *M. tuberculosis *Δ*sigC *mutant infected guinea pigs for long-term survival studies, those monitored to 8 weeks showed weight gain, good grooming and did not appear ill compared with their counterparts infected with the wild type and complemented mutant strains. These data show that the *M. tuberculosis *Δ*sigC *mutant mounts a slower initial rate of growth and is associated with fewer caseating lesions than in wild type. Moreover, these observations suggest that compared to mice, the *M. tuberculosis *Δ*sigC *mutant produces a more delayed initial infection in guinea pigs. Based upon the bacterial growth profiles defined in mice [16,17], this pattern seen after Δ*sigC *mutant infection of guinea pigs is most compatible with the defective growth *in vivo *(*giv*) pattern.

## Discussion

*M. tuberculosis *mutant strains which exhibit the *imp *phenotype and show milder lung pathology as well as prolonged host survival in comparison to the wild type strain have been described [16,18-23]. In our current survival study, gross pathology and histopathology revealed the mild pathology with the Δ*sigC *mutant consistent with this reduced pathology pattern. On the other hand, the lung CFU counts of mice infected with the Δ*sigC *mutant strain, while relatively high, began to lag three weeks post infection and remained between 1 to 1.5 log_10 _units lower than the CDC1551 and Δ*sigC *complemented mutant strains. In contrast to earlier studies of the Δ*sigC *mutant [20], the lung CFU count showed relative containment compared with the wild type; this occurrence could play a role in the relative reductions in some of the inflammatory parameters observed with the mutant. The major cell types infiltrating the alveolar spaces of the CDC1551 and Δ*sigC *complemented mutant strain infected mice were neutrophils, monocytes and lymphocytes. Interestingly, the numbers of infiltrating neutrophils in the BAL of Δ*sigC *mutant infected mice were at least 10-fold less than the other two groups. Reduced levels of TNF-α, IL-1β, IL-6, IL-12, IFN-γ, CCL-2 and CCL-5 were also detected in the lung homogenates of Δ*sigC *mutant infected mice. However, the lung is a separate compartment from the BAL and the levels in one may not necessarily relate to the other.

These cytokines are commonly associated with the inflammatory environment of *M. tuberculosis *infection [3,4,7,9-12,33-37]. We did not examine the role of the adaptive immune system in clearing *M. tuberculosis *infection. However, previous studies have placed a great emphasis on the role of a T helper 1 (Th1) type response, involving the engagement of CD4^+ ^and CD8^+ ^effector T cells and the production of macrophage-activating cytokines like IFN-γ, TNF-α and IL-12. Together with T cells and NK cells, macrophages have been shown to secrete TNF-α, IL-12 and IFN-γ during intracellular infections [38]. IFN-γ is an important mediator of macrophage activation and regulation of this cytokine has been considered crucial in combating infection [8,11]. IL-12, which induced IFN-γ production by lymphoid cells [39,40], did not change significantly in this study. This may be because we focused on the primary site of infection, i.e., the lung tissue homogenates, and not on lymphoid tissues. The lack of neutrophil infiltration and reduced levels of pro-inflammatory cytokines like TNF-α, IL-1β, IL-6 and IFN-γ in the lungs of Δ*sigC *mutant infected mice may explain the milder disease in the DBA/2 and SCID mice. However, in SCID mice a significant difference in cellular infiltration in the BAL was not observed. Since neutrophils have been identified in tuberculous lung granulomas in humans, guinea pigs and mice [41], their role in granuloma formation has been suggested [30,42,43]. Neutrophils have been shown to produce different chemokines which are crucial for bacterial clearance [44,45]. Yet the levels of both CCL-2 and CCL-5 in the lungs of Δ*sigC *mutant infected mice were marginal and might not be involved in the recruitment of neutrophils in this study.

Both IL-1β and IL-6 are involved in the host immune response to *M. tuberculosis *infection and are produced by monocytes, macrophages, dendritic cells and neutrophils [46]. IL-1β was detected in lung sections of tuberculosis patients [47,48] and in the animal models [49,50]. This cytokine has been postulated to play a role in restricting the spread of the bacillary growth after *M. tuberculosis *infection by forming granulomas [51,52]. Our data would support this hypothesis because we saw severe granuloma-like lesions in the lungs of the CDC1551 and Δ*sigC *complemented mutant groups but not among the Δ*sigC *mutant infected mice where a lower concentration of IL-1β was detected.

Guinea pigs form caseating granulomas in their lungs in response to *M. tuberculosis *infection while infected mice do not. In this study, Δ*sigC *mutant infected guinea pigs had undetectable CFU counts 4 weeks after infection in contrast to both the CDC1551 and the Δ*sigC *mutant complemented infected groups. A recent study compared the survival of identical pools of *M. tuberculosis *mutants in both animal species after aerosol infection and found that *M. tuberculosis *genes required for survival were cleared earlier in guinea pigs than in mice [53]. Differences in host response to the invading bacilli may be species specific and the Δ*sigC *mutant strain can express a different phenotype in guinea pigs than in mice. Here we showed that the Δ*sigC *mutant strain proliferated poorly with a *growth in vivo *(*giv*) defect in guinea pigs. A similar finding was also observed when guinea pigs and mice were infected with the *M. tuberculosis *Δ*dosR *mutant strain [54]. Similar findings have been recently reported in a study of several sigma factor mutants evaluated in the guinea pig model [55].

The major findings in this work are the low influx of neutrophils and reduced levels of known pro-inflammatory cytokines which are associated with *M. tuberculosis *infection in the BAL of mice exposed to the Δ*sigC *mutant strain. We selected the DBA/2 mouse strain for our study because of it susceptibility to *M. tuberculosis *infection hence fewer bacilli were required to induce the disease [56]. These mice survived for more than one year after aerosol infection with the Δ*sigC *mutant strain and immunological analysis revealed that the attenuated mutant strain was not as immunogenic as both the wild type and complemented strain. Our data are similar to those in a recent study looking at the immunological changes of susceptible mouse strains to *M. tuberculosis *(H37Rv) infection [13]. In that study, elevated numbers of neutrophils in the BAL correlated with higher susceptibility to succumbing to *M. tuberculosis *infection. However, our study is the first to show that neutrophil infiltration can be affected by an attenuated *M. tuberculosis *mutant strain. Indeed, lipid heterogeneity, rather than genetic variations, is believed to be the major cause of differences in pathogenicity between strains [57,58]. Transcriptional profiling of the Δ*sigC *mutant versus the wild type strain [20] revealed that genes involved in biosynthesis of cofactors, cell wall components, fatty lipids, energy metabolism, protein folding and stabilization, and protein synthesis were downregulated. Hence it will be of interest to investigate whether such changes in the cell envelope may contribute to the expression of the *imp *phenotype of the Δ*sigC *mutant strain in mice.

## Conclusion

We have found that the Δ*sigC *mutant with the *imp *phenotype is unable to trigger a strong early immune response. This may occur due to a low influx of neutrophils and reduced levels of pro-inflammatory cytokines. Our study is the first to show that neutrophil infiltration can be affected by an attenuated *M. tuberculosis *mutant strain.

## Methods

### Bacterial strains and culture conditions

The *sigC *mutant was constructed as described [20]. *M. tuberculosis *CDC1551 wild type, Δ*sigC *deleted mutant and the Δ*sigC *deleted complement mutant were cultured in Middlebrook 7H9 (liquid medium containing 0.05% TWEEN 80 and supplemented with OADC and 0.2% glycerol) at 37°C and 0.5% CO_2_. At the appropriate times OD readings at 600 nm were taken to determine bacterial density.

### Mouse virulence assays

Female DBA/2 and CB17-SCID mice were infected by the aerosol route by placing liquid inoculum into the nebulizer of a Glass-Col aerosol machine and running the standard program. Their lungs were implanted with ~100 bacteria per lung at day 0 of the experiment. At day 1, lungs were collected separately and homogenized and dilutions plated onto 7H10 agar plates to confirm that comparable numbers of bacteria were implanted per mouse lung. The bacteria were incubated at 37°C and the colony forming units (CFU) determined.

### Guinea pig infection

Female outbred Hartley guinea pigs (~500 g) were infected using a Madison aerosol generation chamber calibrated to deliver approximately 50 to 100 bacteria per lung at day 0. Three to five animals were sacrificed 4 and 8 weeks post infection and their entire lungs homogenized for CFU counts.

### Cytokine Assay

Supernatants from homogenized lungs were collected and passed through a 0.22 μM Millipore filter. Using the DuoSet ELISA kits from R&D Systems, (Minneapolis, MN) supernatants were tested for the presence of CCL-2, CCL-5, TNF-α, IL-6, IL-1β, IL-4, IL-12p70 and TGF-β1. IL-10 and IFN-γ sandwich ELISAs were performed using reagents from MabTech (Cincinatti, OH). Cytokine assays were conducted according to the instructions provided by the manufacturers.

### BAL and Cytosmear

Bronchoalveolar lavage cells (BAL) were harvested by exposing the trachea and excising a small incision using a scissor. A blunt needle connected to a 1 ml syringe is inserted into the cut and ice cold PBS is gently flushed into the trachea and lungs. BAL cells were washed twice at 1200 RPM for 7 min at 4°C and resuspended in PBS with 1% FCS. 100 μl of BAL cells was mounted on a glass slide with cytospin centrifugation (StatSpin Cytofuge 2) and sealed. Slides were air dried overnight and stained with Protocol HEMA 3 stain set and differential cell counts performed.

### Histopathology

Lungs were excised from all animals and stored in 10% formalin, embedded and stained with haematoxylin and eosin (H&E) for pathological analysis or with Ziehl-Nielsen stain to evaluate the presence of acid fast bacilli in these organs.

### Statistical analysis

The GraphPad Prism 4 software program was used to perform all statistical analysis (GraphPad Software Inc., San Diego, CA). Kaplan-Meier analysis was used to determine statistical significance of the differences in survival of mice. The statistical significance of three-way comparisons was tested by two-way analysis of variance (ANOVA) with Bonferroni post-tests.

## Authors' contributions

K. Abdul-Majid designed and performed the experiments and data analysis and wrote the paper; L. Ly designed and performed guinea pig experiments; P. Converse and D. Geiman guided experimental design, provided constructs, and assisted in manuscript preparation; D. McMurray designed guinea pig experiments and assisted in manuscript preparation; W. Bishai obtained funding, guided experimental design and assisted in manuscript preparation.
